# Stable Ozonides plus Vitamin E Acetate (Ozoile) for Treatment of Genitourinary Syndrome

**DOI:** 10.3390/medicina60060880

**Published:** 2024-05-27

**Authors:** Carlo Ronsini, Irene Iavarone, Natalino Lacerenza, Giada Andreoli, Maria Giovanna Vastarella, Pasquale De Franciscis, Mario Passaro, Raffaella De Simone, Domenico Giraldi, Rosalia Lizza, Giampaolo Mainini

**Affiliations:** 1 Department of Woman, Child and General and Specialized Surgery, University of Campania “Luigi Vanvitelli”, 81100 Naples, Italy; n.lacerenza@yahoo.com (N.L.); andreoli.giada@gmail.com (G.A.); mariagiovanna.vastarella@unicampania.it (M.G.V.); pasquale.defrancoscis@unicampania.it (P.D.F.); 2 Società Campano Calabro Apulo Lucana di Ginecologia ed Ostetricia (S.C.C.A.L.), 80133 Naples, Italy; ireneiavarone2@gmail.com (I.I.); pm70doc@gmail.com (M.P.); giampaolomainini@libero.it (G.M.); 3 UOC di Ginecologia ed Ostetricia AORN A. Cardarelli, 80131 Naples, Italy; raffaelladesimone@yahoo.it; 4 AORN S. G. Moscati, 83100 Avellino, Italy; domenico.giraldi@gmail.com; 5 UOC di Ginecologia ed Ostetricia PO San Luca, 84078 Vallo della Lucania, Italy; rosalializza@hotmail.it

**Keywords:** genitourinary syndrome, vulvovaginal atrophy, ozonides, vitamin E

## Abstract

*Background and Objectives*: Genitourinary syndrome, previously defined as vulvovaginal atrophy, manifests with signs and symptoms deriving from estrogen diminution in the female genitourinary tract. Stable ozonides are derivatives of artemisinin found to be stable against strong basic and acidic conditions. Vitamin E is an important antioxidant diminishing the output of reactive oxygen species in the oxidation of fats and the emanation of free radicals, reducing cellular injury and aging. The primary aim of the present study was to assess the positive effects of an ozonide plus a vitamin E acetate-based compound (Ozoile) on genitourinary syndrome symptom relief after a maximum of 20 days of treatment. *Materials and Methods*: The inclusion criteria for patients’ enrollment were women of child-bearing age or in menopause reporting genitourinary syndrome’s related symptoms, such as pain, burning, a bad smell, dyspareunia, dryness, itching, bleeding, and nervousness. The exclusion criteria were Sjogren’s syndrome and patients administered retinoic acid, an agent that causes mucosal dryness. Participants completed a questionnaire before and after 20 days of treatment. *Results*: The incidence of pain decreased from 16.7% to 11.8% (*p*-value < 0.0001). In addition, the mean symptom intensity decreased from 2.10 to 0.87 (*p*-value < 0.0001). Dryness was the most frequent pre-treatment symptom and decreased from 85.5% to 53.8% (*p*-value < 0.0001) (mean: 2.21 vs. 0.90; *p*-value < 0.0001). *Conclusions*: Ozoile was effective in reducing most gynecologic symptoms related to genitourinary syndrome. However, further studies are needed to compare its effect with other standards of care.

## 1. Introduction

Genitourinary syndrome, previously defined as vulvovaginal atrophy, manifests with signs and symptoms deriving from estrogen diminution in the female genitourinary tract. This syndrome results in both genital dysfunctions, such as dryness, dyspareunia, burning, and irritation, and urinary dysfunctions, such as dysuria, nocturia, urgency, and recurrent urinary tract infections [1]. Vaginitis is a common condition occurring during women’s lives that can be caused by infective or inflammatory processes or following an alteration in the normal vaginal microbiota [2]. Characteristic symptoms occurring in atrophic vaginitis are vaginal dryness, dysuria, abnormal vaginal discharge, itching, burning, and dyspareunia [3]. Vaginal dryness is a typical issue of women’s lifespan, occurring at all ages, but prevalently after the menopause [4]. Most of women affected by vaginal or vulvovaginal atrophy report suffering from vaginal dryness, as demonstrated in patients administered oral contraceptives [4]. The definition of vulvovaginal atrophy includes a high-occurrence complaint due to the decreased heterogenization of the vaginal tissue, leading to several symptoms, such as vaginal dryness, irritation, soreness, and dyspareunia, associated with frequency, urgency, and urge incontinence, in what can be identified as genitourinary syndrome [5,6,7]. Vulvovaginal atrophy is mostly related to decreased estrogen levels, such as in the menopausal period [5,8]. In the early premenopausal period, there is an incidence of vulvovaginal atrophy of about 4%, while in the late postmenopausal period, this increases up to 47% [5].

Stable ozonides are derivatives of artemisinin found to be stable against strong basic and acidic conditions [9]. 1,2,4-trioxolane (ozonide) has shown several biological activities, such as anti-infective, anti-fungal, anti-inflammatory, anti-cancer, anti-proliferative, and anti-arrhythmic activities [9]. Another treatment method is local vitamin E: vitamin E is an important antioxidant diminishing the output of reactive oxygen species in the oxidation of fats and the emanation of free radicals, reducing cellular injury and aging [10]. These properties allow vitamin E to repair the vaginal epithelium, improving the activity of the mucosal cells in the same context and hence decreasing inflammatory processes [11]. These elements show positive effects on vulvovaginal atrophy [11].

The primary aim of the present study was to assess the positive effects of an ozonide plus a vitamin E acetate-based compound (Ozoile) on genitourinary syndrome symptom relief after 20 days of treatment.

## 2. Materials and Methods

### 2.1. Study Population

The present study was a prospective monocenter interventional cohort analysis conducted from October 2023 to January 2024 at the Department of Woman, Child, and General and Specialized Surgery of the University of Campania “Luigi Vanvitelli” and authorized by the Ethics Committee and the Institutional Review Board of the above-mentioned institution.

The inclusion criteria for patients’ enrollment were women of child-bearing age or in menopause reporting genitourinary syndrome’s related symptoms, such as pain, burning, a bad smell, dyspareunia, dryness, itching, bleeding, and nervousness. The exclusion criteria were Sjogren’s syndrome, any rheumatologic condition leading to mucosal dryness, and patients administered retinoic acid, an agent that causes mucosal dryness. This study complied with the Declaration of Helsinki, and every patient signed an informed consent form to agree to participate in this study. The participants were administered an ozonide plus vitamin E acetate-based compound, registered as Ozoile.

### 2.2. Study Procedures

On the day of the first examination, the participants were asked by a doctor to answer the questions contained in a questionnaire. The questionnaire was composed to detect genitourinary syndrome’s symptoms and was approved by the Institutional Review Board of the institution. Candidates were only identified through the initial letters of their name and surname (to protect their privacy), their age, and their fertility category, which could have been prepubescent, fertile, menopause, or pregnancy. After, the gynecological examination was performed by an experienced gynecologist. Both the date of the first visit and the date of the first vaginal swab—with the relative sample’s result—were assessed. The diagnosis of vaginitis was confirmed or denied. Secondly, the clinician evaluated the presence of dysuria and its intensity. The stages of intensity were mild, moderate, or severe. In addition, the occurrence of the following symptoms was reported: pain, burning, a bad smell, dyspareunia, dryness, itching, bleeding, nervousness, and compliance. Also, the symptoms’ intensity was registered and could be mild, moderate, or severe. The patients were administered a treatment based on Ozoile to be used twice a day (2-to-3 puffs) for 10-to-15 days, and after, it was continued with 1 application per day for 5-to-20 days, depending on the patients’ relief, which concerned the tolerance, stabilization, and disappearance of symptoms.

On the day of the second examination, which occurred 7 ± 3 days after the last day of treatment, the following data were reported in the above-mentioned questionnaire: the date of the examination, the result of the vaginal sample, and, finally, the intensity of symptoms that were mentioned during the first examination. The parameters of evaluation were the same, i.e., mild, moderate, or severe, to establish if there were benefits. Also, eventual pain after the application of Ozoile, and—if present—for how much time it lasted, was assessed, with the possible answers being between initially only, within 15 min, or over 15 min.

The time to improvement from the start of the treatment was also notified, with possible answers being between immediately, within the first 5 days, and after the first 10 days. The duration of treatment varied between 10 and 20 days after the questionnaire was concluded with a symptomatic analysis, the results of which could have been equal, better, or absent compared with the time before the treatment started.

### 2.3. Statistical Analysis

Mean and ranges were used as descriptive parameters of sample characteristics. The intensity of symptomatology was expressed as a parametric scale of values: 0 = absent; 1 = light; 2 = moderate; and 3 = heavy. The treatment time was considered from the day of the first application to the day of the last application and was expressed in days. The incidence of individual events within the sample was reported as a percentage. The Chi2 test compared the frequencies of the observed values. Reported pre- and post-treatment symptom means were compared using Student’s *t*-test for paired dependent variables. Single-symptom outcome means were compared with post-treatment means only in patients who had the symptom at enrollment, with a reported value of ≥1. The Statistical Package for Social Sciences software, version 25.0 (IBM Corporation, Armonk, NY, USA) was adopted for all statistical calculations. For all performed analyses, a *p*-value of <0.05 was considered significant.

## 3. Results

Between May 2023 and December 2023, we treated 186 patients with vaginitis and/or dysuria for genitourinary syndrome at our institution with Ozoile. The mean age of the test sample was 49.3 years. 63.4% of the treated patients were menopausal. All women, before treatment, underwent vaginal swabbing for pathogens. A total of 55 patients (29.5%) had a positive swab result. The two pathogens most commonly present were *E. coli* (12.3%) and *C. albicans* (8.1%). All patients had, at enrollment, at least one of the following symptoms: pain, burning, a bad smell, dyspareunia, dryness, itching, bleeding, or nervousness. The sample characteristics are summarized in Table 1.

### Outcome

**Figure 1:**
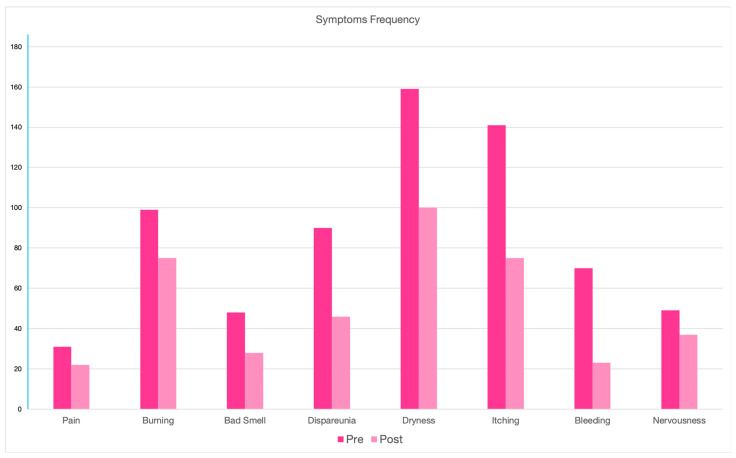
Symptoms’ incidence.

**Figure 2:**
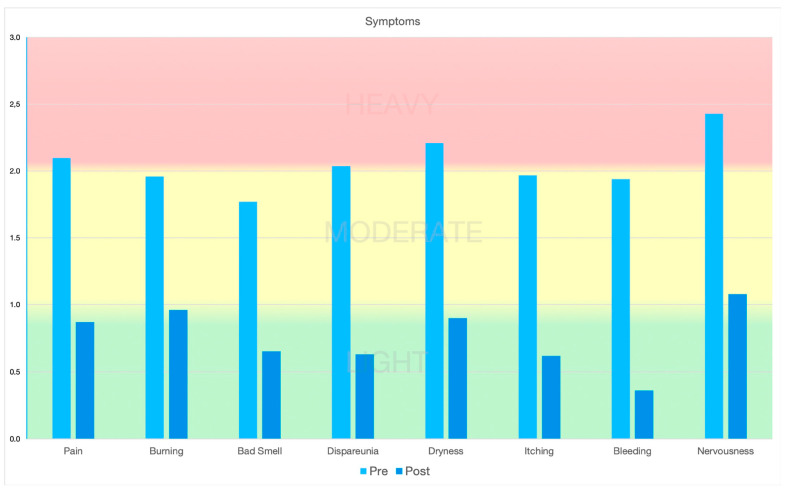
Symptoms’ intensity.

After the application of Ozoile, a reduction in the incidence and intensity of all the symptoms analyzed was observed. The incidence of pain decreased from 16.7% to 11.8% (*p*-value < 0.0001). In addition, the mean symptom intensity decreased from 2.10 to 0.87 (*p*-value < 0.0001). Burning decreased from 53.2% to 40.3% (*p*-value < 0.0001) (mean: 1.96 vs. 0.96; *p*-value < 0.0001). A bad smell was reported by only 28 patients after treatment (15.1%) versus 48 (25.8%) pre-treatment (*p*-value < 0.0001) (mean: 1.77 vs. 0.65; *p*-value < 0.0001). Dyspareunia was reduced from 48.4% to 24.7% (*p*-value < 0.0001) (mean: 2.04 vs. 0.63; *p*-value < 0.0001). Dryness was the most frequent pre-treatment symptom and decreased from 85.5% to 53.8% (*p*-value < 0.0001) (mean 2.21 vs. 0.90; *p*-value < 0.0001). Itching was reduced from 75.8% to 40.3% (*p*-value < 0.0001) (mean: 1.97 vs. 0.62; *p*-value < 0.0001). A total of 70 patients reported blood loss before treatment (37.6%). Only 23 (12.4%) also reported this symptom post-treatment (*p*-value < 0.0001) (mean: 1.94 vs. 0.36; *p*-value < 0.0001). Finally, the feeling of nervousness was reduced from 26.3% to 19.9% (*p*-value < 0.0001) (mean: 2.43 vs. 1.08; *p*-value < 0.0001). All patients underwent a post-treatment vaginal swab. No positivity was found. The clinical outcome data are summarized in Table 2. The evolution of pre- and post-treatment symptomatology is shown in Figure 1 and Figure 2.

A total of 59 patients (32.3%) perceived burning during the vulvovaginal application of Ozoile. Of these, 88.1% reported the symptom only in the immediacy of application. Another 10.2% reported symptom resolution within the first 15 min. Only one case reported discomfort with persistence beyond 15 min. In total, 180 (96.8%) patients felt some degree of relief after treatment. 98.7% of patients achieved the peak of post-treatment relief within 10 days of treatment, and 28 patients (15.1%) reported the complete resolution of symptoms. Of these patients, 10.7% benefited from the immediate resolution of symptoms and 57.1% within the first 5 days of treatment. Treatment was prolonged for no less than 10 days and no more than 20 days, with an average duration of 16.05 days. The data on the treatment modalities are summarized in Table 3.

## 4. Discussion

Genitourinary syndrome has a great impact on patients’ quality of life [12]. The enormous variety of symptoms associated with this syndrome results in different clinical forms of presentation with different degrees of severity. Therefore, the perception of discomfort a woman feels can translate into different symptoms. However, this condition finds a common basis in its manifestations in local irritation due to mucosal atrophy, infection, or inflammation [1]. Moreover, since the vagina is a self-protecting system, the establishment of alterations leading to genitourinary syndrome results in a vicious circle destined to worsen the symptomatology. This condition should not only be understood as one concerning menopausal patients but also, if a loss of vaginal well-being occurs, as one that exposes the woman to a whole series of sequelae. A bacterial imbalance can represent both triggers and consequences. Nearly 30 percent of the patients still in our study had a positive vaginal swab for infection. The well-being of the vaginal microbiota is critical to both maintain the patient’s genital health status and prevent the onset of further disorders, such as cystitis or endometriosis [13,14]. As evidence that infected mucosal quality is a predisposing factor for the development of infections, none of our patients after treatment showed vaginal bacterial imbalance. The same principle also applies to one of the symptoms considered, a bad smell. In our series, Ozoile improved this condition in terms of frequency (25.8% versus 15.1%; *p*-value < 0.0001) and intensity (1.77 vs. 0.65; *p*-value < 0.0001), keeping the symptom, when not completely resolved, well below a tolerable threshold. Another underlying principle of complaints related to genitourinary syndrome is an inflammatory status. Vaginal use of vitamin E is known to be effective in the short-term treatment of genitourinary syndrome [11].

However, the lack of standardization of administration and dosages makes its use still debated. Its administration by stable ozonides, which are mixtures of oxygen and ozone bound to the olefinic bonds of olive oil fatty acids, ensures that vitamin E is delivered vaginally as well as regulating gene transcription by promoting the repair of tissue damage through the activation of signaling functions related to oxidation–reduction reactions bound by ozone and oxygen molecules [15,16]. This mechanism involves an anti-inflammatory effect on the one hand [17] and a microbicidal effect on the other.

The microbicidal effect appears to be broad-spectrum due to the high affinity toward bacterial and fungal wall protein components and the oxidizing action of bioperoxides [18].

The anti-inflammatory effect is related to the inhibition of COX2, with the inhibition of subsequent prostaglandin production. In the orthopedic field, a randomized clinical trial of 180 patients showed that using Ozoile as a topical anti-inflammatory was superior to NSAIDs in controlling joint pain [19].

Previous in vitro studies have demonstrated the microbial action of ozone’s potent ability to decrease the growth of bacteria and mycetes [20].

Therefore, using Ozoile affects both inflammation and bacterial colonization factors that may underlie genitourinary syndrome. Finally, the use of Ozoile also has a documented tissue-remodeling action. Indeed, it activates the synthesis of HIF-1alpha and VEGF, promoting hypoxia resistance and neoangiogenesis [21,22,23,24,25].

The limitations of our study include its observational nature, which limits its comparison with other therapies, such as the topical use of estrogen or hyaluronic acid. The short observational period could also constitute a bias. In addition, the evaluation of the efficacy of Ozoile is based on patient self-reported symptoms, exposing the veracity of the data to a placebo effect. Nevertheless, the high reported efficacy, the size of the sample under investigation, and the solid statistical significance provide an excellent basis for future investigations regarding the use of this preparation in the management of genitourinary syndrome. In addition, 30% of our cohort had positive swabs, and those infections were treated with therapies other than Ozoile. Unfortunately, we do not know the correlation between symptoms and vaginal swab positivity in women of childbearing age and in menopause. It would be interesting to investigate that relationship in further studies.

## 5. Conclusions

The soothing and antimicrobial action of Ozoile was shown to be effective in reducing gynecologic symptoms related to genitourinary syndrome. However, the noncomparative nature of this study makes it useful as a baseline only for future investigations and comparisons with current treatment standards. Future studies focusing on the objectification of tissue response by biopsy specimens and not just the symptomatology reported by patients would be desirable. As interesting as the data on the topical use of vitamin E are, it does not appear to be covered in the major international guidelines [22,23].

## Figures and Tables

**Table 1:** Patients’ characteristics.

186 Patients	Mean (Range) or N (%)
Age	49.3 (16–82)
- Prepubescent	4 (2.2)
- Fertility	64 (34.4)
- Menopause	118 (63.4)
Vaginal sample positivity	55 (29.5)
- *E. coli*	23 (12.3)
- *C. albicans*	15 (8.1)
Vaginitis	36 (19.4)
Dysuria	172 (82.5)
Pain	31 (16.7)
Burning	99 (53.2)
Bad smell	48 (25.8)
Dyspareunia	90 (48.4)
Dryness	159 (85.5)
Itching	141 (75.8)
Bleeding	70 (37.6)
Nervousness	49 (26.3)

**Table 2:** Clinical outcomes and clinical incidence of symptoms. NA = Not applaiable.

**Symptom**	**Mean Pre**	**Mean Post**	***p*** **-Value**
Pain	2.10	0.87	<0.0001
Burning	1.96	0.96	<0.0001
Bad smell	1.77	0.65	<0.0001
Dyspareunia	2.04	0.63	<0.0001
Dryness	2.21	0.90	<0.0001
Itching	1.97	0.62	<0.0001
Bleeding	1.94	0.36	<0.0001
Nervousness	2.43	1.08	<0.0001
**Symptom**	**N (%)**	**N (%)**	***p*** **-Value**
Pain	31 (16.7)	22 (11.8)	<0.0001
Burning	99 (53.2)	75 (40.3)	<0.0001
Bad smell	48 (25.8)	28 (15.1)	<0.0001
Dyspareunia	90 (48.4)	46 (24.7)	<0.0001
Dryness	159 (85.5)	100 (53.8)	<0.0001
Itching	141 (75.8)	75 (40.3)	<0.0001
Bleeding	70 (37.6)	23 (12.4)	<0.0001
Nervousness	49 (26.3)	37 (19.9)	<0.0001
Vaginal sample positivity	55 (29.5)	0 (0)	NA
- *E. coli*	23 (12.3)	0 (0)	
- *C. albicans*	15 (8.1)	0 (0)	

**Table 3:** Treatment outcomes.

	N (%)
Burning at application	59 (32.3)
- Only at application	52 (88.1)
- ≤15 min	6 (10.2)
- >15 min	1 (1.7)
Symptomatology	
- Same	6 (3.2)
- Partial relief	152 (81.7)
- Complete relief	28 (15.1)
Days for best response	
- Instant	5 (2.7)
- ≤5 days	72 (38.7)
- 5 to 10 days	107 (57.5)
- >10 days	2 (1.1)
Days for complete relief	
- Instant	3 (10.7)
- ≤5 days	16 (57.1)
- 5 to 10 days	8 (28.6)
- >10 days	1 (3.6)
Mean treatment duration (days)	16.05 (10–20)

## Data Availability

Data are protected for privacy reasons, they can be shared upon request to the corresponding author.
